# Expansion of health insurance in Moldova and associated improvements in access and reductions in direct payments

**DOI:** 10.7189/jogh.06.020702

**Published:** 2016-12

**Authors:** Thomas Hone, Jarno Habicht, Silviu Domente, Rifat Atun

**Affiliations:** 1 Department of Primary Care and Public Health, Imperial College, London, UK; 2 WHO Country Office in Kyrgyzstan, World Health Organization; 3 WHO Project Office in Greece, World Health Organization; 4 Harvard T. H. Chan School of Public Health, Boston, MA, USA

## Abstract

**Background:**

Moldova is the poorest country in Europe. Economic constraints mean that Moldova faces challenges in protecting individuals from excessive costs, improving population health and securing health system sustainability. The Moldovan government has introduced a state benefit package and expanded health insurance coverage to reduce the burden of health care costs for citizens. This study examines the effects of expanded health insurance by examining factors associated with health insurance coverage, likelihood of incurring out–of–pocket (OOP) payments for medicines or services, and the likelihood of forgoing health care when unwell.

**Methods:**

Using publically available databases and the annual Moldova Household Budgetary Survey, we examine trends in health system financing, health care utilization, health insurance coverage, and costs incurred by individuals for the years 2006–2012. We perform logistic regression to assess the likelihood of having health insurance, incurring a cost for health care, and forgoing health care when ill, controlling for socio–economic and demographic covariates.

**Findings:**

Private expenditure accounted for 55.5% of total health expenditures in 2012. 83.2% of private health expenditures is OOP payments–especially for medicines. Healthcare utilization is in line with EU averages of 6.93 outpatient visits per person. Being uninsured is associated with groups of those aged 25–49 years, the self–employed, unpaid family workers, and the unemployed, although we find lower likelihood of being uninsured for some of these groups over time. Over time, the likelihood of OOP for medicines increased (odds ratio OR = 1.422 in 2012 compared to 2006), but fell for health care services (OR = 0.873 in 2012 compared to 2006). No insurance and being older and male, was associated with increased likelihood of forgoing health care when sick, but we found the likelihood of forgoing health care to be increasing over time (OR = 1.295 in 2012 compared to 2009).

**Conclusions:**

Moldova has achieved improvements in health insurance coverage with reductions in OOP for services, which are modest but are eroded by increasing likelihood of OOP for medicines. Insurance coverage was an important determinant for health care costs incurred by patients and patients forgoing health care. Improvements notwithstanding, there is an unfinished agenda of attaining universal health coverage in Moldova to protect individuals from health care costs.

Moldova is the poorest country in Europe. Following independence from the Soviet Union in 1991, Moldova experienced rapid economic decline and has faced economic challenges since, hampering government efforts aimed at health system strengthening, financial sustainability, and universal health coverage (UHC) [1,2] – the sustainable development goals (SDGs) for health [3].

The economic downturn in Moldova led to health system funding shortages, reductions in service provision, increased out–of–pocket payments for users [4], and a rise in tobacco and alcohol use. Life expectancy at birth fell from 69 years in 1989 to 66 years in 1995, and the health burden from infections (particularly tuberculosis) and chronic illness rose [5,6]. Adverse economic conditions led to emigration, with the resident population of Moldova falling from 3.62 million in 2003 to 3.56 million in 2013 [7]. In 2006, around 20% of the population lived on less than US$ 2 (purchasing power parity) a day [8], while gross domestic product (GDP) per capita was US$ 1967 in 2011–the lowest in geographical Europe [5]. The GDP has increased from US$ 1.3 billion in 2000 to US$ 7.3 billion in 2012 [9], but approximately one quarter comes from remittances from Moldovan population working abroad [8].

The government of Moldova has embarked on health system reforms aimed at rationalising excess hospital capacity [6], reducing service duplication and developing primary health care (PHC) [4] in order to improve health outcomes, provide financial protection and achieve financial sustainability. *The Health Sector Strategy for 1997–2003* [4] set out plans to develop an efficient, effective, responsive and equitable health system [10–12]. Following the 1994 Constitution, which guaranteed a right to health, a state–funded free health service package was introduced in 1999 [4,6], followed by the Law on Mandatory Health Insurance (MHI) in 2001, and the establishment of the National Health Insurance Company (CNAM) in 2004 [4].

The MHI is compulsory for Moldovan citizens–and aims to provide complete insurance coverage–but in reality individuals may choose their own insurance or not to purchase any [4]. Certain individuals (non–working groups including students, children, pensioners, disabled etc.) receive insurance coverage without payment covered by the government [4]. MHI coverage entitles individuals to a benefits package of covered services–including selected primary and secondary care services, emergency care, and dental services. In 2009, primary care services were extended, free of charge, to all irrespective of insurance status, and in 2012, services covered by the benefit package were further extended [4]. Nonetheless, many medicines are not covered and patients incur informal payments due to limited financing of the benefit package [4,13]. The *National Health Policy 2007–2021* [14] was followed by *the Healthcare Development Strategy 2008–2017* [15] which, alongside wider health system reform, specifically aimed to expand insurance coverage through financial incentives and mandating an insurance policy when renewing government issued licenses [4].

Earlier studies highlight the significant burden of OOP incurred for hospital services (mostly due to informal payments) and for medicines [13] (**Box 1**). This study uses routine administrative data on insurance coverage and health care utilization, and household surveys to explore the effect of health system reforms on OOP, and applies robust econometric methods to analyze the likelihood of being uninsured, incurring an OOP for medicines or health services, or forgoing health care by socio–economic and demographic characteristics.

Box 1 **Health System Financing in Moldova**

**Trends in health system financing:** Total health expenditure (THE) as a percentage of GDP rose from 8.1% in 2002 to 11.72% in 2012. While higher than the EU average of 9.61% in 2012, the absolute level of health expenditure per person (PPP$) is the lowest in Europe at US$ 344, compared with the EU average of U$ 3307 [5].

In 2012, health expenditure from public sources was 45.5% of THE compared to the EU average of 76.0%, illustrating the large role private sources play. Public sector expenditure on health as a proportion of total government expenditure rose from 11.7% in 2007 to 13.3% in 2012 – similar to the EU average of 15.2% [5].

The majority of private health expenditures (83.2% in 2012) is OOP and has risen from 79.9% in 2003. Pharmaceutical expenditures accounted for 72% of the OOP payments in 2010 [16].

**Purchaser–provider split:** In 2003, the creation of CNAM introduced a purchaser–provider split in Moldovan health system by separating health financing and service delivery. CNAM is responsible for direct contracting of hospitals and PHC providers, and for 85% of the government expenditure on health [17]. Of this expenditure, around 49% was spent on hospitals, 29% on PHC, 9% on ambulance services, 7% on specialized outpatient care, 4% on compensated outpatient medicines, and 2% on complex health care services (2011 data), with little variation in these proportions since 2007 [17].

**Geographical variation in health system financing:** Healthcare spending across regions (rayons) has been uneven, with per capita funding across rayons in 2003 differing by a factor of 4.6. Urban rayons received a substantially greater share of funds due to concentration of hospital and specialist care. Following financial reorganization in 2004 that centralised pooling of funds with CNAM, in 2010 the difference fell to 3.8 [17].

## METHODS

### Analytical framework

This study uses a health systems framework [16,17] to guide the analysis (**Box 2**). The framework builds on earlier approaches used to analyze health system reforms [18,19]. The national Healthcare Development Strategy 2008*–*2017 follows a similar structure to the health systems framework used in the study, enabling the analysis of the changes in health system goal of financial protection following health system reforms aimed at expanding insurance coverage, and exploring the association of insurance coverage with service utilization, OOP payments incurred and forgone health care.

Box 2 **Health System Framework used for analysis**

**Health system functions:** We identify four key health system functions which the policy makers can modify to achieve health system goals: (i) **governance and organization** – the policy and regulatory environment, stewardship function of the ministry of health and its relationship with other levels of the health system, and structural arrangements for insurers/purchasers, health care providers and market regulators; (ii) **financing** – how the funds are collected, funds and risks pooled, finances allocated within the health system and how health care providers are remunerated; (iii) **resource management** – how physical, human and intellectual resources are generated and allocated, including their geographic and needs–based allocation; and (iv) **service delivery** that includes both public health services and individual health services provided within the community, PHC, hospitals, and other health institutions. Health services are produced using governance and organization, financing and resource management functions.

**Health system objectives:** We define four objectives which the policy makers need to balance in relation to individual and public health services: **equity** (including access and use of services by different population groups), **efficiency** (efficient allocation of resources to right interventions and producing them at low cost), **effectiveness** (the extent to which interventions provided are evidence based and safe) and **responsiveness** (of care providers to user needs, including choice of providers).

**Health system goals:** There are three health system goals in our framework which the system aims to achieve. The first goal is **health**, both the level and distribution of population health as measured by morbidity and mortality. The second goal is **financial protection**, for which we examin the level and distribution of health expenditures (targeting of health insurance), levels of health insurance coverage, and levels of financial protection (out of pocket expenditures, and catastrophic health expenditures) for different population segments. The third goal is **user satisfaction**, specifically the satisfaction of the population with the health system.

### Data sources

Two main sources of data were used for the years 2006–2012. First, publically available datasets from the Moldovan National Center for Health Management (CNMS) were collected. CNMS collates data from public health care provider reports. We extracted health service utilization information relating to number of hospitalisations, average number of visits per person, and emergency calls per 1000 residents.

Second, the monthly Household Budget Survey (HBS) was used. The HBS is based on an internationally validated survey and is undertaken by the Moldovan National Bureau of Statistics (NBS) [7]. The HBS is nationally representative and is undertaken through two–stage sampling based on regional areas and a random selection of households. Approximately 5500 households (15 000 individuals) are surveyed annually on a wide range of questions relating to the economic situation of the household and individuals. Responses for the years 2006–2012 were obtained from the NBS. We selected questions relevant to this analysis including demographic, socio–economic, health and health care–related questions. Our outcome variables of interest were calculated from survey questions: “If individuals currently have health insurance”; “if individuals paid for any service (inpatient or outpatient) either formally or informally when using care in the last four weeks”, and “if you were unwell in the last four weeks, but did not use healthcare”. Because of issues of non–response (up to 40% in some years), the age and gender distribution of the HBS was compared to national population data (from NBS [7]) showing high similarity.

### Analysis

Using the health system analytical framework [14,15], we examine elements of financial protection in the context of the health system objectives. Equity is a key health system objective for this analysis. We examine equity in insurance status, OOP payments, and foregone health across demographic groups. We demonstrate the interactions between insurance status and equity in other financial protection elements such as OOP. Additionally, we also explore the health system objectives of responsiveness in terms of preferred health care provider, effectiveness through forgone health care, and efficiency in terms of national utilization trends. We triangulate these findings to understand how factors contributing to financial protection are being met.

### Descriptive analysis

CNMS data on health care utilization are shown over time. Individual responses on preferred of health care provider, for those with health care use in the last four weeks, were stratified by provider and year.

For insurance coverage, CNMS data was compared with individual HBS responses (stratified by employed status and age groups) over time.

Insurance coverage trends were compared between individual HBS responses and from administrative data (CNMS) sources. Furthermore, we stratified the insurance status of the respondents in the HBS by occupation and age group.

The percentages of individuals reporting OOP payments were described by consultative services, inpatient services and drugs, and by each year. Mean incurred costs by individuals were shown. Additionally, mean costs were compared to average monthly earnings.

### Logistic regression

We employed logistic regression to calculate the likelihood of: being uninsured, incurring an OOP for medicines or any health care service from health care used in the last four weeks, and not using (foregoing) health care when reporting a health problem in the last four weeks. Logistic regression was employed as the most appropriate method for binary outcomes.

Covariates from the HBS survey were used to control for and highlight explanatory factors. We included in all models: age group (0–17 years, 18–24, 25–34, 35–49, 50–59, 60–74 and 75+); gender; chronic disease status (yes or no); employment status (employed, self–employed (non–agriculture), self–employed (agriculture), unpaid family worker, unemployed (including those not of working age); and educational attainment (pre–school or no education, primary, secondary, and college or university). We also included year (2006–2012) to look for time trends.

For the regressions on the likelihood of an OOP and forgone health care, we included disability (yes or no) and uninsured (yes or no) as covariates. Additionally, for the regressions on the likelihood of an OOP we included first choice of health care provider (family doctor’s office, home visit, polyclinic (health center), hospital or other (eg, pharmacy). For the regression of foregone health care, we only examined the years 2009–2012, as the question was not in earlier surveys. Analyses were carried out at the individual level, with adjustments for the clustered nature of the survey. All individual responses were included for analyses, except the likelihood of being uninsured. The regression was restricted to those aged over 18 and under the age of 60 years, as individuals outside these ages are eligible for free insurance coverage. We report adjusted odds ratios and 95% confidence intervals. Interaction terms between covariates and year (linear trends) were additionally tested.

## RESULTS

**Table 1** shows the descriptive statistics, which categorises the respondents by socio–economic and demographic covariates, and additionally overall responses to key variables of interest.

**Table 1:** Numbers and percentage distribution of Household Budget Survey (HBS) respondents by year (2006–2012), socio–economics and demographics, and key variables of interest

	2006		2007		2008		2009		2010		2011		2012	
**No.**	**%**	**No.**	**%**	**No.**	**%**	**No.**	**%**	**No.**	**%**	**No.**	**%**	**No.**	**%**
**Age group (years):**														
0–17	4560	28.2	4507	27.2	4383	26.7	3974	26.4	3703	25.8	3577	24.4	3424	24.5
18–24	1488	9.2	1626	9.8	1539	9.4	1509	10.0	1303	9.1	1319	9.0	1203	8.6
25–34	1771	11.0	1871	11.3	1780	10.8	1684	11.2	1607	11.2	1728	11.8	1589	11.4
35–49	3299	20.4	3186	19.2	3152	19.2	2870	19.1	2702	18.8	2627	17.9	2467	17.7
50–59	2311	14.3	2514	15.2	2601	15.8	2345	15.6	2273	15.8	2440	16.7	2352	16.8
60–74	2027	12.5	2063	12.4	2124	12.9	1910	12.7	2072	14.4	2151	14.7	2174	15.6
75+	707	4.4	822	5.0	841	5.1	774	5.1	719	5.0	817	5.6	765	5.5
**Sex:**														
Male	7477	46.3	7666	46.2	7514	45.8	6960	46.2	6692	46.5	6720	45.8	6428	46.0
Female	8686	53.7	8923	53.8	8906	54.2	8106	53.8	7687	53.5	7939	54.2	7546	54.0
**Chronic condition:**														
Yes	3861	23.9	4112	24.8	4303	26.2	3993	26.5	4253	29.6	4038	27.6	4241	30.4
No	12 302	76.1	12477	75.2	12109	73.8	11073	73.5	10126	70.4	10621	72.5	9733	69.7
**Employment status:**														
Employed	4339	26.9	4525	27.3	4540	25.5	4061	25.2	3849	25.0	3987	27.2	3681	26.3
Self–employed non–agriculture	516	3.2	428	2.6	475	2.7	473	2.9	440	2.9	526	3.6	480	3.4
Self–employed agriculture	2661	16.5	3048	18.4	2892	16.2	3037	18.9	3393	22.1	4095	27.9	4339	31.1
Unpaid family worker	350	2.2	354	2.1	242	1.4	252	1.6	244	1.6	295	2.0	288	2.1
Unemployed	8297	51.3	8234	49.6	9671	54.3	8265	51.4	7453	48.5	5756	39.3	5186	37.1
**Education:**														
Pre–school or none	2365	14.6	2378	14.3	3682	20.7	3140	19.5	3039	19.8	2043	13.9	1943	13.9
Primary	2351	14.6	2307	13.9	2253	12.6	1965	12.2	1750	11.4	1711	11.7	1516	10.9
Secondary	8249	51.0	8503	51.3	8497	47.7	7971	49.6	7735	50.3	7832	53.4	7705	55.1
College or university	3198	19.8	3401	20.5	3388	19.0	3012	18.7	2855	18.6	3073	21.0	2810	20.1
**Disabled:**														
No	15 303	94.7	15 676	94.5	15 519	87.1	14 135	87.9	13 501	87.8	13 710	93.5	13 027	93.2
Yes	860	5.3	913	5.5	2301	12.9	1953	12.1	1878	12.2	949	6.5	947	6.8
**Uninsured:**														
No	12 390	76.7	12 656	76.3	12 718	71.4	11 368	70.7	10 879	70.7	11 116	75.8	10 593	75.8
Yes	3773	23.3	3933	23.7	5102	28.6	4720	29.3	4500	29.3	3543	24.2	3381	24.2
**First choice provider:**														
Home visit	157	6.6	137	6.0	210	10.7	251	11.0	220	9.5	251	10.3	219	8.9
Family doctor office	792	33.3	656	28.7	479	24.4	665	29.2	633	27.4	633	26.0	799	32.6
Polyclinic health center†	1235	52.0	1302	57.0	1071	54.5	1084	47.6	1185	51.3	1201	49.3	1176	48.0
Hospital	185	7.8	185	8.1	202	10.3	216	9.5	193	8.4	181	7.4	127	5.2
Other (eg, pharmacy	7	0.3	5	0.2	3	0.2	62	2.7	77	3.3	172	7.1	128	5.2
**Sought health care in last 4 weeks:**														
Yes	2376	14.7	2285	13.8	1965	13.0	2278	15.1	2308	16.1	2438	16.6	2449	17.5
No	13 787	85.3	14304	86.2	13 202	87.0	12 788	84.9	12 071	84.0	12 221	83.4	11 525	82.5
**Any out–of–pocket for drugs:**														
No	11 692	72.3	12 003	72.4	13 116	73.6	11 610	72.2	10 797	70.2	10 245	69.9	9584	68.6
Yes	4471	27.7	4586	27.6	4704	26.4	4478	27.8	4582	29.8	4414	30.1	4390	31.4
**Any out–of–pocket for services:**														
No	15 632	96.7	16 097	97.0	17 199	96.5	15 626	97.1	14 935	97.1	14280	97.4	13562	97.1
Yes	531	3.3	492	3.0	621	3.5	462	2.9	444	2.9	379	2.6	412	3.0
Foregone health care*:														
No	–	–	–	–	–	–	2190	63.7	1607	57.0	2376	62.1	2333	57.4
Yes	–	–	–	–	–	–	1250	36.3	1214	43.0	1453	38.0	1733	42.6
Number of respondents (N)	16 163		16 589		17 820		16 088		15 379		14 659		13 974	

*This question was not asked in the years prior to 2009.

†Polyclinics include family doctors and out–patient specialists, especially in larger cities and urban centers.

### Service utilization

We examined average health care utilization rate at the national level and preferred first contact provider from HBS respondents. The health care utilization rate at the national level rose between 2006 and 2012 (**Table 2**), with the hospitalization rate (per 100 residents) increasing from 16.7 in 2006 to 18.4 in 2012 and the average number of outpatient visits per person rising from 6.02 to 6.45. These numbers are broadly in line with European averages (of 18.04 hospitalisations and 6.93 outpatient visits per person in 2012) [5].

**Table 2:** Healthcare utilization (2006–2012) [20]*

	2006	2007	2008	2009	2010	2011	2012
Hospitalizations per 100 residents	16.7	17.2	17.8	18.0	18.1	18.5	18.4
Average number of visits per person	6.0	6.2	6.3	6.3	6.5	6.4	6.5
Emergency calls per 1000 residents	266.3	281.4	282.7	301.9	282.7	279.5	271.1

*Includes responses from individuals who sought health care in the last four weeks and their first preferred health care provider. Those seeking care at a hospital are likely be underestimated due the infrequency of events and small samples employed. Polyclinics include family doctors and out–patient specialists, especially in larger cities and urban centers.

**Figure 1:**
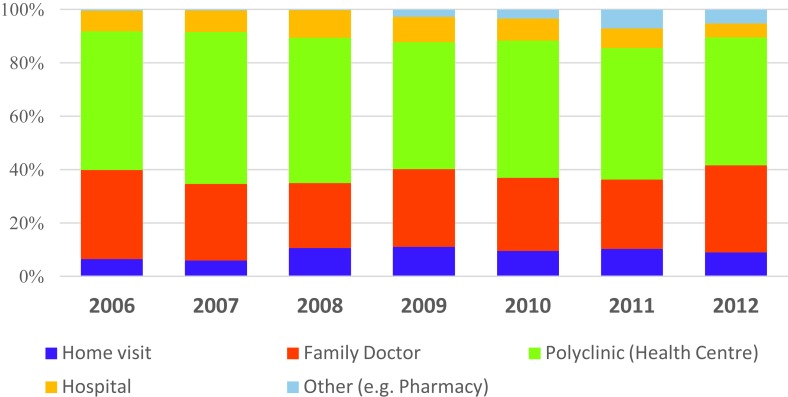
Choice of health care provider for individuals who sought medical care in the last four weeks (2006-12). Shows responses from individuals who sought healthcare in the last four weeks and their first preferred healthcare provider. Those seeking care at a hospital are likely underestimated due to the infrequency of events and small samples employed. Polyclinics include family doctors and out–patient specialists, especially in larger cities and urban centres.

Responses in the HBS from individuals who sought health care in the last four weeks show the majority sought health care in former polyclinics (centers that in majority of cases include both family doctors and out–patient specialists) (48.0% in 2012) with family doctor offices (32.6%), home visits (8.9%) and hospitals (5.2%). These trends have remained fairly constant since 2003 (**Figure 1**).

### Insurance coverage

Approximately 75% of HBS respondents reported having health insurance during the period 2006–2012 (**Table 3**). National statistics suggest rising coverage of health insurance from 76.1% to 80.3%. In 2012, only 60% of the working age population (18–59 years) had insurance coverage, with the self–employed and those working in family business having lower coverage levels: 24% of self–employed agriculture workers, 57.8% of self–employed non–agriculture workers, and 53.8% of unpaid family workers had insurance. In 2012, the main reasons for being uninsured were unemployment (27.9%), cost (26.2%), “they would pay for healthcare anyway” (13.9%), working informally (13.1%), “belief that it would not be needed” (8.5%) and working abroad (8.1%).

**Table 3:** Insurance coverage national and occupational groups (2006–2012) [7,20]

	2006	2007	2008	2009	2010	2011	2012
From the National Household Budget Survey (individuals):							
National coverage	76.7	76.3	77.5	75.5	75.7	75.8	75.8
By occupation:							
- Employed	83.3	85.7	87.1	85.2	84.6	84.9	84.1
- Self–employed (non–agriculture)	19.8	17.1	20.2	18.8	22.3	22.6	24.0
- Self–employed (agriculture)	32.1	37.3	39.1	41.3	45.7	54.7	57.8
- Unpaid family worker	37.4	37.3	41.3	41.3	52.9	51.9	53.8
- Unemployed*	92.7	90.3	76.9	78.2	78.4	90.7	91.1
By age group (years):							
0–18	98.3	99.2	98.8	98.5	98.7	98.4	98.2
18–24	62.8	59.7	64.5	59.4	61.5	58.6	58.9
25–34	50.3	48.4	50.0	48.8	47.5	51.6	46.8
35–49	56.1	55.5	57.0	53.9	53.5	52.9	54.6
50–60	66.4	67.1	69.0	66.8	66.7	67. 6	66.6
60–74	98.1	98.5	97.6	96.9	95.6	96.7	96.7
75+	100.0	100.0	99.9	99.7	99.9	99.9	99.6
From administrative data sources:							
National coverage	76.1	76.2	77.7	80.0	78.6	79.3	80.3

*Includes those not of working age.

The odds of not having health insurance were examined using multivariate logistic regression (**Table 4**). Age was an important determinant of being uninsured, with those aged 25–43 and 35–49 years of age, respectively, 2.9 (OR = 2.898, *P* < 0.001) and 2.3 times (OR = 2.261, *P* < 0.001) more likely to be uninsured than those aged 18–24 or 50–60 years of age.

**Table 4:** Multivariate regression results on likelihood of being uninsured for individuals aged 18–60 years (2006–2012)*

	AOR	*P*–value	95% CI
**Age group: **			
18–24 (Ref)	1.000		
25–34	2.898	0.001	2.682–3.130
35–49	2.261	0.001	2.102–2.433
50–60	1.033	0.409	0.957–1.115
**Sex:**			
Male (Ref)	1.000		
Female	0.599	0.001	0.575–0.623
**Chronic disease:**			
No (Ref)	1.000		
Yes	0.303	0.001	0.285–0.323
**Employment status:**			
Employed (Ref)	1.000		
Self–employed (non–agriculture)	24.339	0.001	21.729–27.262
Self–employed (agriculture)	27.381	0.001	25.631–29.249
Unpaid family worker	23.781	0.001	20.583–27.476
Unemployed	4.915	0.001	4.590–5.262
**Education:**			
Pre–school or none (Ref)	1.000		
Primary	7.170	0.001	4.170–12.327
Secondary	6.047	0.001	3.866–9.459
College or university	3.182	0.001	2.029–4.990
**Year:**			
2006 (Ref)	1.000		
2007	1.108	0.016	1.019–1.204
2008	0.997	0.949	0.916–1.085
2009	1.079	0.088	0.989–1.177
2010	1.008	0.860	0.921–1.103
2011	0.933	0.133	0.852–1.021
2012	0.920	0.074	0.840–1.008
No.	59 151		

AOR – Adjusted Odds Ratio, 95% CI – confidence intervals

*P < 0.001. Cluster robust standard errors used.

Females were less likely to be uninsured than men (OR = 0.599, *P* < 0.001), as were those with chronic health conditions (OR = 0.303, *P* < 0.001). All categories of employment were substantially more likely to be uninsured than those employed. The self–employed, and unpaid family workers were all more than 20 times more likely to be uninsured. The unemployed were 5 times more likely (OR = 4.915, *P* < 0.001). All categories of education were more likely to be uninsured than those with only pre–school or no–education with primary, secondary, and college or university educated individuals 7.7, 6.1 and 3.2 times more likely to be uninsured.

Over time, there was no evidence of a trend in change in likelihood of being uninsured. Models with interactions with time (results not shown) suggest that the likelihood of being uninsured declined over time for those with chronic conditions (compared to those without), females (compared to males), and the self–employed (agriculture) and unpaid family workers (compared to employed). There was an increased likelihood in being uninsured for the unemployed over time (compared to other employment categories).

### Private and out–of–pocket expenditure

Around 15% of HBS respondents who sought health care reported a payment for consultative services and 2% for inpatient care in 2012 (**Table 5**) compared with 20% and 3% in 2006 respectively. The average costs for both consultative services and medicines had increased since 2006.

**Table 5:** Percentage of individuals reporting costs for health care services and average costs incurred (2006–12) [7]

	2006	2007	2008	2009	2010	2011	2012
Of those who sought health care in the last 4 weeks, the percentage incurring costs (formal and informal) for:							
Consultative services*	20.3%	19.7%	23.9%	18.4%	16.9%	13.8%	14.9%
Inpatient services	2.9%	3.4%	4.7%	2.7%	3.1%	2.2%	2.3%
Drugs	82.6%	83.6%	84.1%	86.5%	88.3%	91.1%	89.2%
Average costs incurred (in Moldovan Lei) for:							
Consultative services	132	161	169	190	195	249	215
Inpatient services	711	1548	1132	1284	1065	2343	833
Drugs	175	221	254	258	290	271	269

*Consultation services include: consultations, analyses, diagnoses, treatments, physiotherapies and medical examinations; Inpatient services including treatment, admission, advice, analysis, interventions, surgery. The results include total costs for formal and informal payment (for consultative and inpatient services). Only small numbers of individuals used hospital services or reported inpatient costs meaning there may be variation across years.

The factors affecting the likelihood of an OOP payment for both medicines and health care services were analyzed separately using multivariate logistic regression (**Table 6**).

**Table 6:** Odds of incurring out–of–pocket expenditure for either drugs or health care services for those who sought medical care in last four weeks*

	Drugs			Healthcare services		
	**AOR**	***P*–Value**	**95% CI**	**AOR**	***P*–Value**	**95% CI**
**Age group (years):**						
0–17 (Ref)	1.000			1.000		
18–24	0.732	0.016	0.568–0.943	1.432	0.006	1.107–1.854
25–34	0.747	0.030	0.575–0.972	1.256	0.081	0.972–1.622
35–49	1.133	0.338	0.878–1.463	1.112	0.396	0.870–1.420
50–59	1.575	0.001	1.219–2.036	0.818	0.106	0.640–1.044
60–74	2.074	0.001	1.650–2.607	0.564	0.001	0.450–0.708
75+	1.976	0.001	1.513–2.579	0.336	0.001	0.252–0.448
**Sex:**						
Male (Ref)	1.000			1.000		
Female	1.133	0.013	1.026–1.251	1.083	0.081	0.990–1.185
**Chronic disease:**						
No (Ref)	1.000			1.000		
Yes	1.911	0.001	1.691–2.160	1.296	0.001	1.160–1.448
**Employment status:**						
Employed (Ref)	1.000			1.000		
Self–employed non–agriculture	0.936	0.734	0.641–1.368	0.999	0.993	0.736–1.355
Self–employed (agriculture)	0.867	0.118	0.725–1.037	0.884	0.112	0.759–1.029
Unpaid family worker	0.610	0.006	0.427–0.871	1.016	0.929	0.717–1.440
Unemployed	0.811	0.013	0.689–0.956	0.903	0.155	0.785–1.039
**Education:**						
Pre–school or none (Ref)	1.000			1.000		
Primary	0.671	0.001	0.551–0.818	1.112	0.345	0.893–1.385
Secondary	0.708	0.002	0.571–0.877	1.238	0.066	0.986–1.555
College or university	0.817	0.104	0.640–1.042	1.391	0.008	1.088–1.777
**Disabled**	**1.066**	**0.478**	**0.893–1.274**	**0.634**	**0.001**	**0.542–0.741**
**Uninsured**	**1.297**	**0.004**	**1.088–1.546**	**3.833**	**0.001**	**3.307–4.443**
**First contact health care provider:**						
Family doctor office (Ref)	1.000			1.000		
Home visit	1.021	0.848	0.824–1.265	0.415	0.001	0.324–0.531
Polyclinic (health center)	0.820	0.056	0.668–1.005	2.723	0.001	2.192–3.381
Hospital	0.722	0.010	0.563–0.926	7.205	0.001	5.667–9.161
Other (eg, pharmacy)	11.400	0.001	4.595–28.282	0.081	0.001	0.034–0.191
**Year:**						
2006 (Ref)	1.000			1.000		
2007	1.041	0.624	0.886–1.223	0.870	0.101	0.737–1.027
2008	1.071	0.432	0.902–1.271	1.266	0.005	1.072–1.495
2009	1.226	0.019	1.034–1.454	0.922	0.335	0.782–1.087
2010	1.418	0.001	1.191–1.687	0.866	0.093	0.732–1.024
2011	1.772	0.001	1.469–2.138	0.707	0.001	0.594–0.842
2012	1.422	0.001	1.189–1.700	0.873	0.116	0.736–1.034
N	16 099			16 099		

AOR – adjusted odds ratio, 95% CI – 95% confidence intervals

*P < 0.001. Cluster robust standard errors used.

Although low numbers and fluctuations in reported costs make conclusions difficult, there appears to be only modest reductions in costs incurred relative to average monthly earnings over time (**Table 7**).

**Table 7:** Average reported health care costs compared to average monthly earnings [7]*

	2006	2007	2008	2009	2010	2011	2012
Average monthly earnings (Lei)†	1697	2065	2530	2748	2972	3042	3386
Comparison of average reported health care costs as a proportion of average monthly earnings:							
Consultative services	7.8%	7.8%	6.7%	6.9%	6.6%	8.2%	6.4%
Inpatient services	41.9%	75.0%	44.7%	46.7%	35.8%	77.0%	24.6%
Drugs	10.3%	10.7%	10.1%	9.4%	9.8%	8.9%	8.0%

*Caution is needed in interpretation due to low numbers reporting inpatient costs.

†Monthly earnings include any earning from salaries and employment, and other forms of income, averaged over all employees. It is adjusted for annual inflation.

### Out–of–pocket payment for drugs

Older individuals (aged over 50 years), who sought health care in the last four weeks, were more likely to incur a cost than those aged under 34 years. Females were 13% more likely to pay (OR = 1.133, *P* = 0.013), while those with chronic conditions were nearly 1.9 times as likely to incurring a cost (OR = 1.911 *P* < 0.001).

Unpaid family workers and the unemployed were less likely to incur costs for medicines, as were those with primary and secondary education. Those without health insurance were 1.3 times more likely (OR = 1.297, *P* = 0.004) to incur an OOP for medicines compared to than those with insurance. Individuals were only less likely to incur a cost for medicines at hospitals or other services (such as pharmacies).

Over the period 2006–2012, the odds of incurring a cost for medicines have increased (up to 1.8 times more likely to incur a cost in 2011 (OR = 1.772, *P* < 0.001) compared to 2006. The only significant interaction with time (results not shown) was insurance status with those without insurance increasingly more likely to pay for medicines OOP over time.

### Out–of–pocket payment for health care services

Individuals who sought medical care in the last four weeks and incurred OOP payments (informally or formally) for consultative or inpatient services showed different trends to costs for drugs. Older individuals and those under 18 were least likely to incur a cost than those aged 18–24. Those with chronic conditions were 1.3 times more likely to pay (OR = 1.296, *P* < 0.001), but those with disability were nearly 40% less likely to pay (OR = 0.634, *P* < 0.001).

Uninsured individuals were 3.8 times (OR = 3833, *P* < 0.001) more likely to incur an OOP payment for health services. There was no difference between employment categories while only those with college or university education were more likely to pay (OR = 1.391, *P* = 0.008).

Those using hospitals and polyclinics respectively were 7.2 times (OR = 7.205, *P* < 0.001) and 2.8 times (OR = 2.723, *P* < 0.001) more likely to incur a cost than at family doctor offices. Home visits were less likely to incur a cost than family doctor office (OR = 0.415, *P* < 0.001).

There was a general decline in the likelihood of incurring an OOP expenditure cost for services, although the difference was not significant every year. The only interaction with time (results not shown) was age with those aged 50–60 years having an increased likelihood of incurring a cost over time compared to other ages.

### Foregone health care utilization

Using multivariate logistic regression the likelihood of not visiting a health care provider when being sick in the last four weeks was examined (**Table 8**). Older individuals were more likely not to seek health care with those 75 years or more 2.4 times (OR = 2.379, *P* < 0.001) more likely than 0–24 year–olds. Females were 9.8% less likely to forego care (OR = 0.902, *P* = 0.003).

**Table 8:** Results from multivariate logistic regression on odds of not utilizing health care when unwell*

	AOR	*P*–Value	CI
**Age group (years):**			
0–17 (Ref)	1.000		
18–24	1.267	0.063	0.987–1.626
25–34	1.342	0.016	1.056–1.704
35–49	1.680	0.001	1.358–2.078
50–59	1.878	0.001	1.521–2.318
60–74	2.008	0.001	1.656–2.435
75+	2.379	0.001	1.972–2.872
**Sex:**			
Male (Ref)	1.000		
Female	0.902	0.003	0.841–0.966
Chronic disease?:			
No (Ref)	1.000		
Yes	1.059	0.244	0.962–1.165
**Employment status:**			
Employed (Ref)	1.000		
Self–employed non–agriculture	0.967	0.803	0.742–1.260
Self–employed (agriculture)	0.793	0.001	0.706–0.891
Unpaid family worker	0.716	0.037	0.522–0.981
Unemployed	0.859	0.014	0.761–0.969
**Education:**			
Pre–school or none (Ref)	1.000		
Primary	1.156	0.104	0.970–1.378
Secondary	0.989	0.907	0.825–1.186
College or university	0.875	0.180	0.719–1.064
**Disabled**	**0.819**	**0.001**	**0.732–0.916**
**Uninsured**	**1.680**	**0.001**	**1.483–1.902**
**Year:**			
2009 (Ref)	1.000		
2010	1.320	0.001	1.174–1.484
2011	1.070	0.231	0.958–1.195
2012	1.295	0.001	1.163–1.443
N	14 156		

AOR – adjusted odds ratio, 95% CI – 95% confidence intervals

*P < 0.001. Cluster robust standard errors used.

Employed and self–employed (non–agriculture) individuals were most likely to not seek health care when sick. The disabled were less likely to forego health care when sick (OR = 0.819, *P* < 0.001). Those without insurance were 1.7 times (68%) more likely not to seek health care when sick (OR = 1.680, *P* < 0.001). 

Over time there was an increasingly likelihood of forgoing health care when sick with individuals on average 1.3 times (30%) more likely in 2012 than 2009. There were no significant interactions between covariates and time suggesting the increasing trend in likelihood of foregoing health care applies to all individuals.

## DISCUSSION

Our results indicate that aspects of financial protection in Moldova are improving, albeit slowly, but with different trajectories for certain demographic groups.

Health service utilization has gradually increased from to 6.45 visits per person in 2012–now in line with the European average of 6.93–and while the majority of individuals use some form of PHC (former polyclinics or family doctor offices), there has been little change over time in utilization levels for PHC services. The inefficient hospital network [21] needs to be rationalised to encourage greater use of PHC services and to develop a more efficient and effective health system.

The challenging economic environment is slowing efforts to improve financial protection–particularly in terms of equity. Our finding that insurance coverage is still not universal–impacting accessibility of services–particularly in older individuals, unemployed, agriculture workers and those of working age is in line with earlier studies [22]. Our findings illustrate that despite the government efforts to expand coverage through financial incentives to purchasing coverage and as a requirement during certain license renewals, not enough progress has been made.

Limited public finances for health are misallocated due to slow progress in tackling the inefficient hospital network which reinforces inequitable provision of health care services [21], and limits the ability of the government to further incentivise insurance coverage and expand the benefit package. Although overall there was no significant improvement over time in insurance coverage, the finding that particular groups–the self–employed (agriculture) and unpaid family workers–had reduced likelihood of being uninsured suggests attempts to expand coverage to these groups are having some success.

Our findings confirm earlier studies [13] that reveal the large burden OOP payments–particularly for medicines. Our results indicate the likelihood of incurring a payment has increased over time for medicines, but declined for health care services. Notably insurance status is a strong determinant of likelihood of incurring an OOP payment. The fact that many medicines are not covered by the MHI package [4] is clearly contributing to OOP burdens. The high average cost for drugs, which has both increased since 2006, is resulting in the unaffordability of medicines and high prices in both the public and private sectors [23]. Financial protection is still not being met in terms of access to medicines, but on the other hand, we see that efforts to curtail informal payments and reduce OOP in services have had some success. Earlier studies in Moldova suggest that cost as a reason for not seeking care when experiencing a health problem has fallen, but is still a major factor especially in poorer income groups [13].

The finding that health care is still being foregone suggests the minor improvements for some groups in insurance coverage and OOP for services, are not translating to improvements in health care utilization. Individuals are increasingly likely to forego health care–notably the uninsured, older, and certain groups of employed individuals. Challenges in expanding insurance coverage are clearly impacting on health care utilization, but health care utilization is also likely to be affected by the limited benefit package, health service quality and non–financial barriers to access [4]. Evidence indicates health insurance is key to protecting individuals against OOP payments and promoting health care utilization [24,25], although for those in the informal sector the effects may be weaker [26].

### Strengths and limitations

The HBS only provides limited insight into financial protection in Moldova over time. As a survey, certain populations–eg, traveling communities or homeless–will be not represented. The high non–response rate ( ~ 40%) raises the issue of reliability of the survey, although the concordance of results with earlier studies and representativeness of age and sex distributions could mean the impact of the high non–response rate is low. There are also potential issues of recall or selective reporting possible. There are limitations in the questions asked in the survey. For example, we do not know individuals’ utilization patterns of health care–only if they have used a health care provider in the last four weeks. Healthcare utilization could confound the results reported here. None–the–less, while we must acknowledge certain groups (eg, those with chronic conditions, older people or the disabled) are likely to use health care more, true financial protection should be equitable across groups. Further research into the exact costs and fees incurred by individuals and explanatory demographic variables is also needed.

Other data sources employed–including CNMS–are from administrative sources that may be prone to errors and issue of data quality. Additionally, as administrative data are only from public facilities, this study is not able to analyze private providers in the health system.

The analyses undertaken may also introduce potential errors. Logistic regression identifies associations through predictive probabilities between groups and outcomes, but cannot prove causality. Our results must be interpreted with this limitation in mind. Additionally, assumptions about grouping of outcomes–including OOP from informal and formal sources and between different services–may obscure finer trends and patterns, but due to small sample size it was not possible to analyze these subgroups separately. Even so, we use multiple covariates not to just examine potential inequities between demographic groups, but also to control for potential effects and elucidate clearer associations than descriptive trends. This allows us to identify whether it is age or employment status, for example, which is the stronger determinant of an outcome. Furthermore, we take into account the clustered nature of the survey design further strengthening the validity of our findings.

## CONCLUSIONS

There is clear evidence that many elements of financial protection are not being met in Moldova. While in some areas–insurance coverage and OOP for services–there is slow improvement, but the increase in OOP for medicines counteracts the improvements observed. Healthcare is potentially being foregone due to limited protection from costs. Progress toward UHC is an integral to the SDGs [3], and removing financial barriers to access is key to attaining UHC. To reduce financial access barriers the Moldovan government should focus on three areas: further expansion of health insurance coverage, tackling costs of medicines and health care services, and improving the efficiency of health system financing. The first, further expansion of insurance coverage and access to services centers, can be achieved by further targeting of coverage to the uninsured, by utilizing incentives (both financial and using legal requirements), and by streamlining the enrolment process [27]. Our results indicate that these efforts in the past may have had only small gains, and marginal returns may be low. Alternatively, building upon the expansion and free entitlement to primary care services for all in 2009, insurance coverage could be extended free of charge maintaining inputs from taxation and insurance premiums. Moldova must weigh up the marginal costs of targeted insurance premiums vs expanding free entitlement on the pathway to UHC.

Second, costs for medicines and services could be reduced by: i) introducing regulations to prevent informal payments and to regularise formal cost–sharing [28]; ii) increasing, through allocated funding and legal powers, the powers of the Moldovan Medicines and Medical Devices Agency (MMDA) and CNAM, in procuring medicines, negotiating prices, and regulating quality, to reduce costs for those who purchase drugs and the cost burden of medicines in the benefit package [23,29]; iii) strengthening the provision of PHC, where the majority of health needs can be dealt with cost–effectively, in resource allocations for both services and medicines [28].

Third, health system financing trends indicate that although Moldova commits a proportion of public sources to health in line with EU averages, the actual amount is very low. The government fiscal space for increased funding of health system could be improved by: (i) increasing tobacco and alcohol taxes–which will not only reduce consumption and tackle the health burden of these substances, but generate revenues for the government, (ii) optimizing and consolidating the inefficient hospital sector (iii) investing in new infrastructure using EU development financing and private sector funding to improve health system efficiency.
